# Different pathogenic mechanisms of early-onset preeclampsia, late-onset preeclampsia, and hemolysis, elevated liver enzymes, low platelet syndrome

**DOI:** 10.4274/tjod.galenos.2019.18559

**Published:** 2020-04-06

**Authors:** Giovanni Sisti

**Affiliations:** 1 Lincoln Medical and Mental Health Center, Department of Obstetrics and Gynecology, New York, United States of America

**Keywords:** Preeclampsia, cbc, netrophils, wbc, prediction, hellp syndrome

## To the Editor,

We read with interest the article titled “First trimester complete blood cell (CBC) indices in early and late-onset preeclampsia” by Örgül et al.^(1)^ published in the Turkish Journal of Obstetrics and Gynecology in June 2019.

We share the same enthusiasm in the use of a cheap and simple CBC count as an early predictor of poor obstetric outcomes. CBC is the first laboratory investigation performed in every pregnant woman and its value is not limited to diagnosing current medical conditions, it can also be used as a predictor of future events.

In their article, Örgül et al.^(1)^ show that white blood cell (WBCs) and neutrophil counts are significantly elevated in the first trimester of pregnancies with early and late-onset preeclampsia, compared with controls. They also give an excellent clinical tool in finding a specific cut-off value, using receiver operating characteristic curve analysis; specifically, 9.55x10^3^/uL for WBCs and 6.45x10^3^/uL for neutrophils.

Unfortunately, in their statistical analysis, when comparing the three groups, they did not perform a post-hoc analysis to compare one group with each other.

In our recent studies, we analyzed CBC indices in pregnancy affected by hemolysis, elevated liver enzymes, low platelet (HELLP) syndrome vs. controls^(2,3)^. We found no differences in the first trimester in terms of neutrophil count, and we did not analyze the total WBC count.^(2)^ We obtained informed consent from the patients included in our study.

We think that the difference between our study and the study of Örgül et al.^(1)^ is due to the different pathogenesis of HELLP syndrome and preeclampsia. We think that preeclampsia is caused by an early placentation defect, whereas HELLP syndrome is determined by a maternal immunologic “storm” of circulating inflammatory molecules triggered in the third trimester.

Örgül et al.^(1)^ mentioned that early-onset preeclampsia was caused by early placentation defects, and late-onset preeclampsia was more related to maternal characteristics: in this regards, as mentioned earlier, a post-hoc analysis would have been very useful.
